# Control strategies for rice “straighthead” through physicochemical and biological methods on arsenic transformation and transportation

**DOI:** 10.3389/fpls.2025.1602704

**Published:** 2025-08-07

**Authors:** Xiaobai Li, Xuhao Pan, Dandan Zhang, Biaolin Hu, Wengui Yan

**Affiliations:** ^1^ Rice Research Institute, Jiangxi Academy of Agricultural Sciences/National Engineering Center for Rice (Nanchang), Nanchang, China; ^2^ Institute of Horticulture, Zhejiang Academy of Agricultural Sciences, Hangzhou, China; ^3^ Tobacco Research Institute, Chinese Academy of Agricultural Sciences, Qingdao, China; ^4^ Retired, Stuttgart, AK, United States

**Keywords:** straighthead, arsenic toxicity, fertilizer, minerals, draining and drying, resistant cultivar, QTL and genes

## Abstract

Straighthead is a widespread physiological disease affecting rice, characterized by sterile florets and distorted palea and lemma, which can reduce grain yield by up to 100%. In recent decades, arsenic (As) has emerged as a focal point in straighthead research. This paper elucidates the relationship between As toxicity and straighthead while reviewing preventive measures, including water and fertilizer management and the application of resistant cultivars. The optimization of water and fertilizer management enhances the redox potential for As oxidation and/or changes the microbial community involved in As demethylation in rice fields, leading to increased immobility or affinity of As with other minerals. Furthermore, we integrate our previous genetic studies on straighthead with the As metabolism to uncover its genetic foundations. The results indicate that quantitative trait loci (QTL) associated with straighthead co-locate with QTL/genes related to As within the rice genome. These QTL/genes are frequently involved in the phosphate/silicate (Pi/Si) transporter responsible for As uptake. Such co-localizations imply that the Pi/Si transporter facilitates the translocation of As from roots to shoots, thereby contributing to the occurrence of straighthead. Throughout this text, we underscore the preeminence of the genetic strategy as an optimal solution for managing straighthead. The adoption of resistant cultivars effectively tackles the multifaceted challenges related to water management, such as high costs, water resource wastage, and potential yield losses. Additionally, it addresses concerns regarding fertilizer application, which is heavily reliant on soil conditions and poses significant environmental pollution risks.

## 1 Straighthead and its history

**Figure 1:**
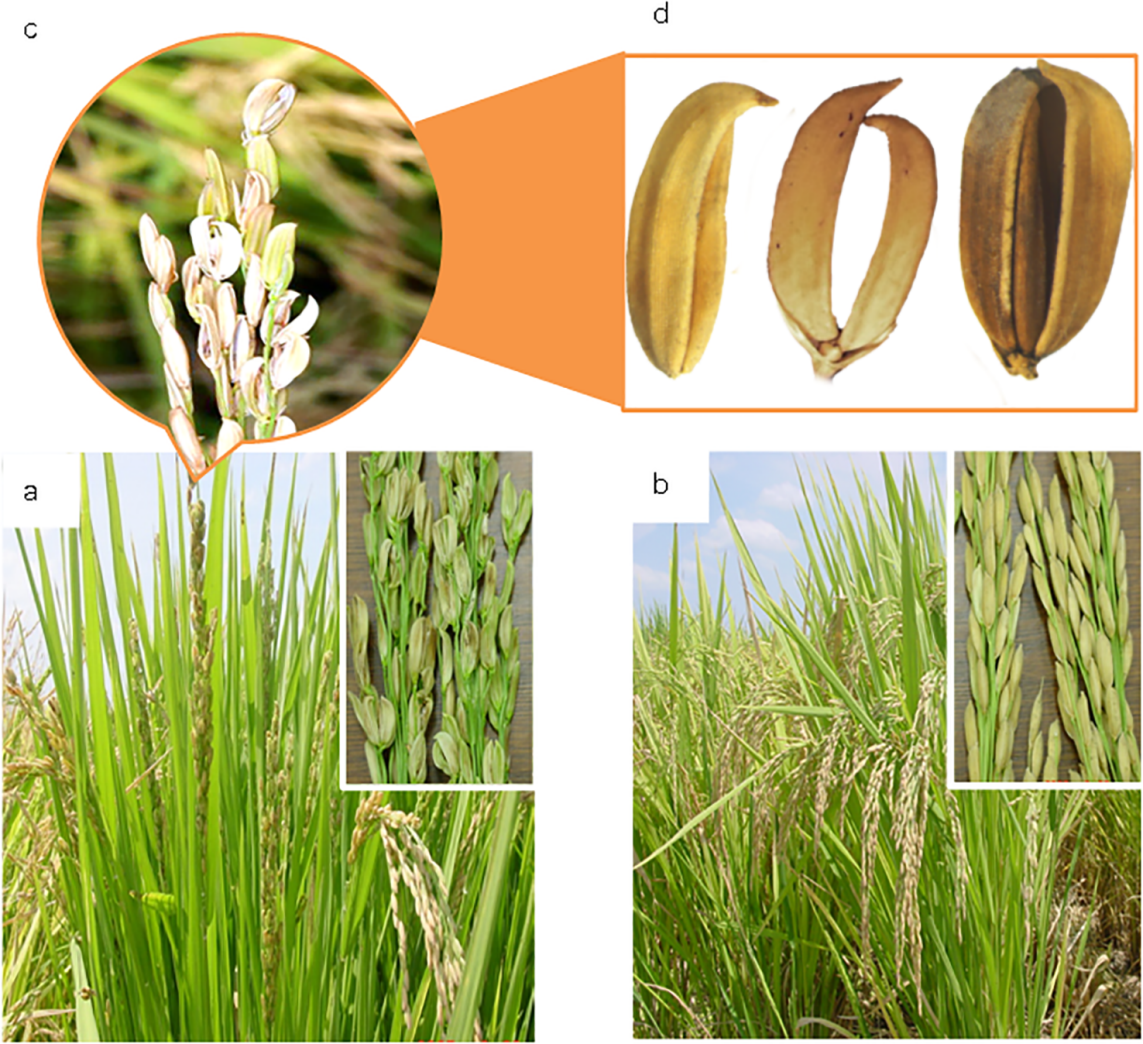
Panicle and kernel morphology of rice plant affected by straighthead. **(a)** Erect panicles and sterile kernels of straighthead susceptible cultivar R312, **(b)** Drooping panicles and fully filled kernels of straighthead resistant cultivar Zhe733; **(c)** Partial-erect panicle with distorted and unfilled kernels and sterile kernel with **(d)** Distorted lemma and palea like a crescent or parrot breaking.

Rice straighthead is a physiological disease that severely affects grain yield. Typical symptoms are sterile florets and distorted palea and lemma. The distorted palea and lemma look like crescent or “parrot breaking” (**Figure 1**). In the early stage (vegetative period), plants with straighthead do not appear stunted or shriveled but instead look quite healthy and are even darker green in color than normal. In the late stage (reproductive period), the panicles of severely infected plants are hard to emerge, and their florets are distorted. The panicles remain erect in the field because the deformed florets set no seeds with no weight, which is why the disease is called “straighthead” in the United States. This lack of visible disease symptoms until heading time makes it very difficult to control, which results in large-scale outbreaks and massive losses. In the field, the affected plants with few or no filled grains could range from several square meters to many hectares. In severely affected areas, the panicles do not emerge out of the sheaths at all, causing almost 100% yield loss.

The first report of straighthead dates back to 1912 in the southern states of the United States, including Arkansas, Louisiana, Mississippi, and Texas (Collier, 1912). The subsequent reports are from Portugal (called “branca”) (Cunha and Baptista, 1958), Japan (Iwamoto, 1969), Thailand (Weerapat, 1979), Australia (Dunn et al., 2006), Bangladesh (Panaullah et al., 2009), and so forth. In China, straighthead in rice is reported much later than most other countries, but it is found in large areas of the middle and lower reaches of the Yangtze River (Chen et al., 2019; Tang et al., 2020). This disease may affect a wider geographic area than presently documented in the literature, because it is possibly known by other names in other rice-producing countries and less attention is paid to this disease than to those caused by fungi, bacteria, and viruses.

Previous studies have revealed that many factors are associated with straighthead, and soil texture is one of the main causes. In the southern United States, straighthead usually occurs on silt and sandy loam soils, which have poor drainage and a low oxygen level during flooding. In Japan, straighthead commonly occurs in paddy fields converted from upland fields, where organic matter, for example, crop straw, has been heavily added (Kataoka et al., 1983), and it is oddly observed on loam or clay soil (Takeoka et al., 1990). In Australia, it has been reported across different soil types, such as self-mulching clays and red loams (Batten et al., 2006). The floret morphology in straighthead is associated with some minerals in the soil, such as boron (B), nitrogen (N), phosphate (Pi), potassium (K), free iron (Fe), arsenic (As), calcium (Ca), manganese (Mn), zinc (Zn), copper (Cu), magnesium (Mg), sulfur (S), and so forth (Belefant-Miller and Beaty, 2007). Increased N application is not just positively correlated with grain yield (Williams, 2003) but also specifically reduces the sterility in straighthead-infected plants (Batten et al., 2006). Rice is apt to exhibit straighthead in soil with low Zn, Cu, Ca, Mg, and Mn, as well as a low pH (Belefant-Miller and Beaty, 2007). In summary, rice straighthead, with a long history of global occurrence across diverse soil types, is influenced by multiple factors, including soil texture and mineral composition, highlighting the need for integrated management strategies to mitigate yield losses.

## 2 Arsenic toxicity and straighthead

### 2.1 Arsenic species and their toxicities

Arsenic (As) has received much attention in recent decades and has taken center stage in straighthead studies. Two species of As exist in soil–water environments: inorganic and organic. Inorganic species (iAs) include arsenate (AsV) and arsenite (AsIII), while organic ones refer to methylated As, for example, Monosodium methanearsonate (MSMA, CH_4_AsO_3_•Na) that is the sodium salt of monomethylarsonic acid (MMA), Dimethylarsinic acid sodium salt (MSDS, C2H_6_AsO_2_•Na) that is the sodium salt of dimethylarsenic acid (DMA). In soil–plant systems, two inorganic forms of AsV and AsIII are inter-convertible in response to environmental conditions, that is, redox potential (Eh) and pH. AsV predominates under aerobic conditions and is highly stable and readily adsorbed to soil components such as hydroxides of metals, organic matter, and clays. AsIII predominates under anaerobic conditions and is 60 times more mobile, soluble, and toxic than AsV, as the former has been shown to react with sulfhydryl (–SH) groups of proteins and enzymes, culminating in the inhibition of cellular function and, eventually, cell death (Abbas et al., 2018; Pandey et al., 2017). Moreover, AsIII can be methylated by soil microorganisms with adenosylmethyltransferase (arsM) to produce various methylated forms, for example, MMA and DMA.

Both inorganic and organic As induce straighthead directly or indirectly, but they differ in bioavailability and biotoxicity (Tang et al., 2016). Widespread straighthead is most likely inclined in As epidemic areas due to iAs accumulation from underground irrigation water. In South and Southeast Asia, irrigation with iAs-rich underground water has increased iAs concentrations in agricultural soil and caused a high incidence of straighthead in rice (Panaullah et al., 2009). In a controlled environment, the application of iAs (AsV) has successfully induced straighthead in rice, whose severity increases essentially with the concentration of As in the soil (Rahman et al., 2008). AsV interferes with Pi metabolism, while AsIII binds to vicinal sulphydryl groups of proteins, thus affecting their structures and/or catalytic functions (Finnegan and Chen, 2012). Additionally, iAs could be transformed into organic As, which also pose a high level of toxicity to plants.

Organic As is derived from widely used herbicides, cotton defoliants, or biotransformation products by soil microorganisms. In rice and Arabidopsis, a large variety of methylated As is detected in the reproductive tissues, while most iAs are found in the vegetative tissues. This suggests that methylated As is more toxic than iAs to the reproductive tissues (Tang et al., 2016; Zheng et al., 2013). The higher toxicity of methylated As to the reproductive system than iAs can partly, but not wholly, be explained by its higher accumulation and more efficient transportation into seeds (Carey et al., 2010; Li et al., 2009a). DMA may interfere with the normal development and function of reproductive organs by disrupting the structural integrity of cell walls, which are crucial for cell expansion, division, and communication in plants (Tang et al., 2020). In hydroponic experiments, DMA application induces abnormal development of rice florets and makes plants exhibit the typical symptoms of straighthead, for example, distorted hulls and unfilled grains (Zheng et al., 2013). In the southern states of the United States, rice straighthead has been frequently observed in flooded fields with previous application of As herbicides such as MMA during cotton rotations, and the highly accumulated MMA has been found in rice grains (Wells and Gilmour, 1977). MMA treatment has been officially regarded as one of the most important criteria for evaluating straighthead.

Methylated As could be produced from the soil, where microorganisms sequentially reduce AsV into AsIII and methylate AsIII into DMA through the transient intermediate MMA by *S*-arsM in many microbial genomes (Qin et al., 2006). However, arsM does not guarantee that a microorganism will actively facilitate As methylation in the presence of the metalloid. This is due to the fact that efficient microbial As efflux by an efflux pump gene (acr3) prevents intracellular accumulation, which in turn represses As methylation (Viacava et al., 2020). Additionally, some fermentative, sulfate-reducing, and denitrifying bacteria can reduce DMA(V) to DMA(III) (Chen et al., 2023). Rice can take up methylated As but not methylate iAs to MMA or DMA (Lomax et al., 2012), although a putative methyltransferase in rice is upregulated in response to As stress (Norton et al., 2008). Methylated As is only found in the shoots of soil-grown rice but not in the filled grains of hydroponic-cultured rice (Lomax et al., 2012). Rice can take up MMA(V) or DMA(V) and reduce MMA(V) to MMA(III) but does not convert it to DMA(V). In contrast, transgenic rice that expresses a bacterial arsM is endowed with the ability to methylate As to mainly DMA, with the consequence of straighthead (Tang et al., 2020). Recently, it has been found that rice can reduce DMA(V) to DMA(III) and DMA(III) is much more toxic to the protoplasts than DMA(V) (Chen et al., 2023). Together, methylated As from pesticides and transformations by microorganisms are important causal agents that induce rice straighthead disease.

### 2.2 Arsenic absorption and transportation

Rice plants take up different forms of As from the soil in the order As (III) > As (V) > DMA (V) > MMA (V) (de Oliveira et al., 2018). Numerous studies show that rice uptakes inorganic As much more efficiently than organic As (Zhao et al., 2013). For example, the efficiency of uptake for AsV is five times higher than for DMA and 2.5 times higher than for MMA (Abbas et al., 2018). However, only iAsIII and DMAsV are identified in the milled (polished) rice grain; the latter is the main species (Pillai et al., 2010). The methylated As is translocated to plant tissues more efficiently and faster than inorganic species through the formation of a reduced complex with glutathione (GSH) or phytochelatins (PC) in roots (Abbas et al., 2018). DMA can be readily translocated via xylem and phloem from root to shoot and grains than other As species in rice (Abedi and Mojiri, 2020; Carey et al., 2010). DMA is more readily permeable into our consumed part (endosperm) than other forms of As (Carey et al., 2010; Zheng et al., 2013), so polished rice (mainly endosperm) has a more significant proportion of DMA than the bran surface (Lombi et al., 2009). AsIII readily accumulates in the ovular vasculature on the surface of the grain, which is responsible for the transportation of water and minerals into the grain (Carey et al., 2010; Lombi et al., 2009). DMA that preferentially accumulates in the caryopsis is highly toxic to the reproductive tissues, so it primarily results in yield loss, whereas AsIII, mainly accumulating in the vegetative tissues, has less effect on yield than DMA (Zheng et al., 2013). The distribution of As species determines their effect on plant growth and reproduction.

As species and bioavailability heavily depend on soil pH. In porewaters of paddy soil, inorganic As is the dominant form with oxyanions of AsIII or AsV, but organic As species are generally present at lower concentrations with the most common forms of MMA(V) and DMA(V) (Keller et al., 2014). Under oxidizing conditions, arsenic acid (H_3_AsO_4_) is a dominant species at low pH (<2), whereas H_2_AsO_4_^−^ and HAsO_4_^2−^ are predominant at pH ranging from 2 to 11 (Bissen and Frimmel, 2003; Smedley and Kinniburgh, 2002). When reduction conditions develop, H_3_AsO_3_ is converted into H_2_AsO_3_^−^ at low pH, but HAsO_3_^2−^ at high pH levels (>12). The high pH induces negative surface charges to promote the desorption of AsIII and AsV into the soil solutions (Ahmed et al., 2011).

**Figure 2:**
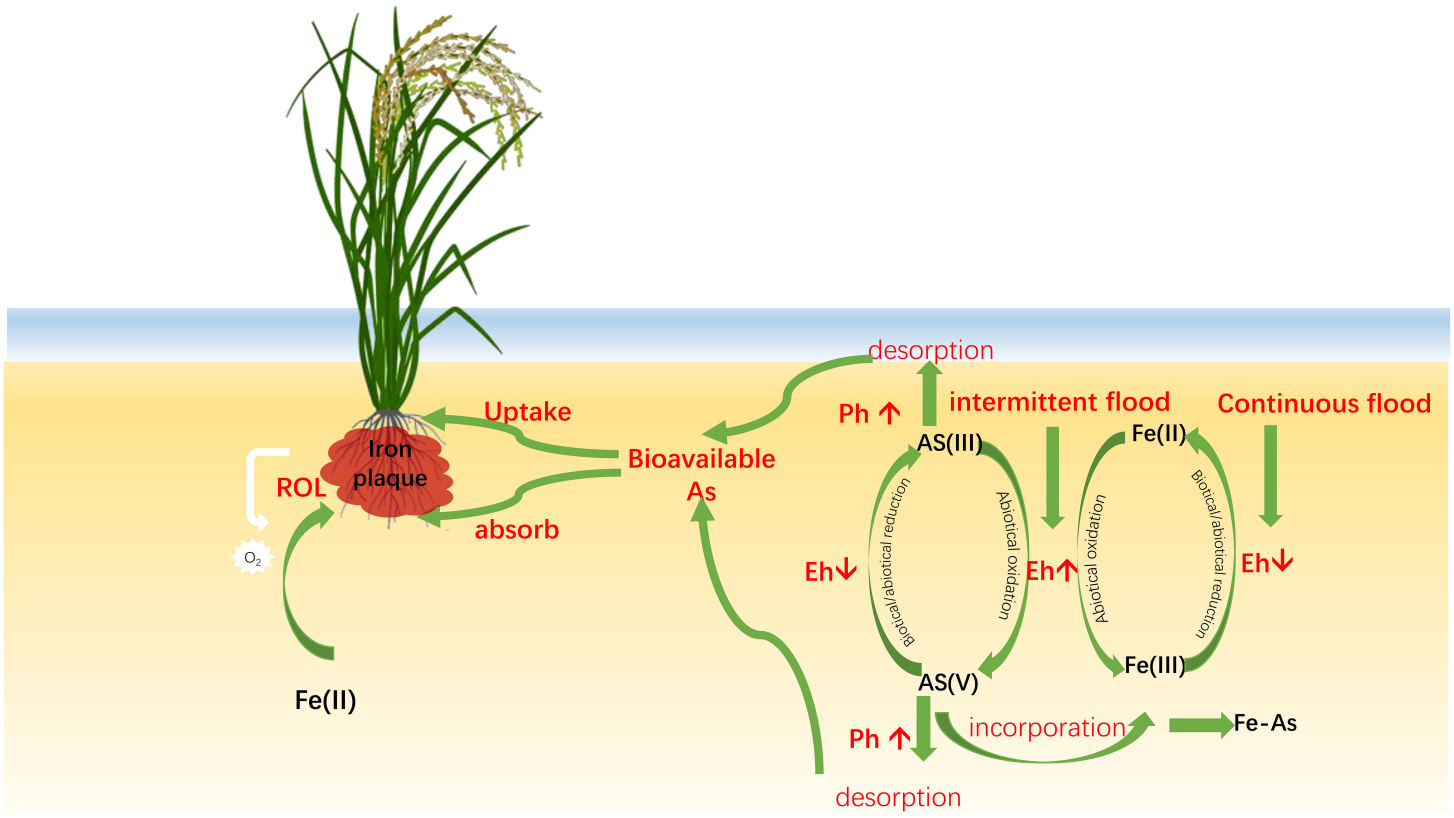
Arsenic cycle coupling with iron (Fe) cycle influenced by redox potential (Eh) and Ph (pH). Under anaerobic condition (continuous flood; low Eh), As is released when FeIII is reduced to FeII as a consequence of a rapid consumption of rice radial oxygen O_2_ (ROL loss) or by iron-reducing bacteria (FeRB). Some species of FeRB and SRB are capable to reduce iAsV to iAsIII. Under aerobic condition (intermitted flood, high Eh), the Fe-plaques on the root surface from the precipitate of Fe-oxides absorbs AsIII for its oxidation to AsV. High pH induces negative surface charges and promotes the desorption of AsIII and AsV into the soil solutions.

Influenced by Eh, As bioavailability is coupled with the Fe-biocycle, including the reductive dissolution of Fe hydro(oxides), the formation of root Fe plaques, and microbial-mediated reduction and oxidation. Fe^2+^ in the rhizosphere is oxidized into oxyhydroxide plaque on the root surface of rice plants. Fe plaques in soil and solution have a very strong binding affinity for As. Thus, Fe plaques contribute to the sequestration and adsorption of As, which take place by radial oxygen loss (Chen et al., 2005; Deng et al., 2010). The Fe-biocycle influences the bioavailability of As through coprecipitation and/or adsorption to FeIII minerals (**Figure 2**) (Chen et al., 2008, 2005). Under anaerobic conditions (low Eh), As is released when FeIII is reduced to FeII due to the rapid consumption of residual O_2_. In contrast, rice roots with larger specific surface area tend to form more Fe-plaques under aerobic conditions by affecting As-uptake kinetics (**Figure 2**). Plaque formation affects the distribution of As and Pi, as well as other heavy metals. AsIII has a high affinity for Fe-plaque and works with FeIII to generate highly insoluble iron AsV (Shakoor et al., 2019). The presence of Fe plaque influences As uptake kinetics by altering the As uptake curves: It changes from linear to hyperbolic for As(III) and from hyperbolic to an S-curve for As(V) (Deng et al., 2010).

## 3 Cultivation strategies to control straighthead

### 3.1 Water management

Water management, referring to the “Draining and Drying” (D&D) practice, has been widely adopted in the southern United States since the early 1900s (Collier, 1912; Tisdale and Jenkins, 1921), which is the only recommended practice to prevent straighthead in rice through the DD50 Computerized Program managed by the agricultural extension system in the United States (Wilson et al., 2010). Rice fields are drained about 2 weeks after a permanent flood, dried thoroughly until cracks appear in the soil and rice leaves begin to curl and exhibit yellowing as drought stress symptoms, and then re-flooded for the remainder of the season. The drying must be completed within 10 to 14 days before the internode elongation starts (Wells and Gilmour, 1977), and the best timing for each farmer to apply the drying is calculated by the online DD50 Program (http://dd50.uaex.edu/dd50Logon.asp). This practice manipulates the water regime in rice paddies by shifting from traditional continuous flooding (permanent flooding) to alternating draining and drying (intermittent flooding). Numerous studies (Fernandez-Baca et al., 2020; Makino et al., 2016; Norton et al., 2017; Yang et al., 2017) have demonstrated that intermittent flooding reduces As concentration in rice grains and shoots, prevents straighthead, and increases grain yield compared to continuous flooding.

The underlying mechanism is involved in the transformation and accumulation of methylated As in soil by microbes. In a field experiment treated with MSMA (MMA), the continuous flooding for straighthead-susceptible cultivars reduced a grain yield by more than 89%, and their grains had significantly higher As content than those in the intermittent flooding (Hua et al., 2013). Prolonged flooding fosters anaerobic soil conditions, which enhance the activity of iron-reducing bacteria (FeRB) and sulfate-reducing bacteria (SRB) (Somenahally et al., 2011a, 2011). These microbes facilitate the reduction of AsV to more mobile AsIII and the methylation of inorganic As (iAs) to DMA, a toxic metabolite strongly linked to straighthead disease. Under continuous flooding, the dissolved Fe²^+^ from iron oxide reduction releases surface-bound As and promotes DMA formation, increasing As bioavailability and uptake by rice roots (Arao et al., 2009; Xu et al., 2008).

In contrast, D&D practice enhances soil aeration during the drying phase, elevating soil redox potential (Eh) and shifting conditions from reductive to oxidative. For example, the soil Eh in intermittent flooding plots (339 ± 84 mV) was more than double that of continuous flooding plots (160 ± 52 mV) (Hua et al., 2013). The oxidative environment suppresses FeRB and SRB activity, reducing the conversion of iAs to DMA and the release of soluble AsIII. Additionally, under aerobic conditions, iron forms insoluble ferric (hydr)oxides (FeIII), which strongly adsorb As V and immobilize soil As, decreasing its availability for plant uptake (Yamaguchi et al., 2011) (**Figure 2**). Concurrently, the drying practice improves soil aeration for rice to grow, thus reducing As uptake due to the decrease of root-specific surface area (RSA) (Deng et al., 2010). Root hairs are vital for water and nutrient absorption and serve as critical sites for increasing RSA. A reduction in RSA, especially in root hairs, diminishes the root–soil contact area, thereby impairing the plant’s ability to absorb As. Another study also demonstrates that oxygen nanobubbles significantly reduce the accumulation of As in rice and mitigate the toxic effects of As on rice (Huang et al., 2023). Thus, D&D-induced aerobic conditions inhibit microbial-mediated methylation of As, reduce soluble As species through FeIII adsorption, and decrease root uptake efficiency, collectively lowering As (especially DMA) accumulation in rice tissues and mitigating straighthead disease.

### 3.2 Sulfur fertilizer

Plants greatly demand sulfur during vegetative growth and seed development because it involves many physiologic processes, for example, photosynthesis, energy generation, photoprotection, and metabolic reactions (Borpatragohain et al., 2019). Also, sulfur plays a crucial role in the translocation and accumulation of As in rice by synthesizing GSH and PCs. GSH, a low molecular weight thiol, is the precursor of PCs, but rice does not produce much GSH. PCs are complex heavy metals, and the sequestered GSH-metal complexes exist in vacuoles (Ashraf et al., 2017).

**Figure 3:**
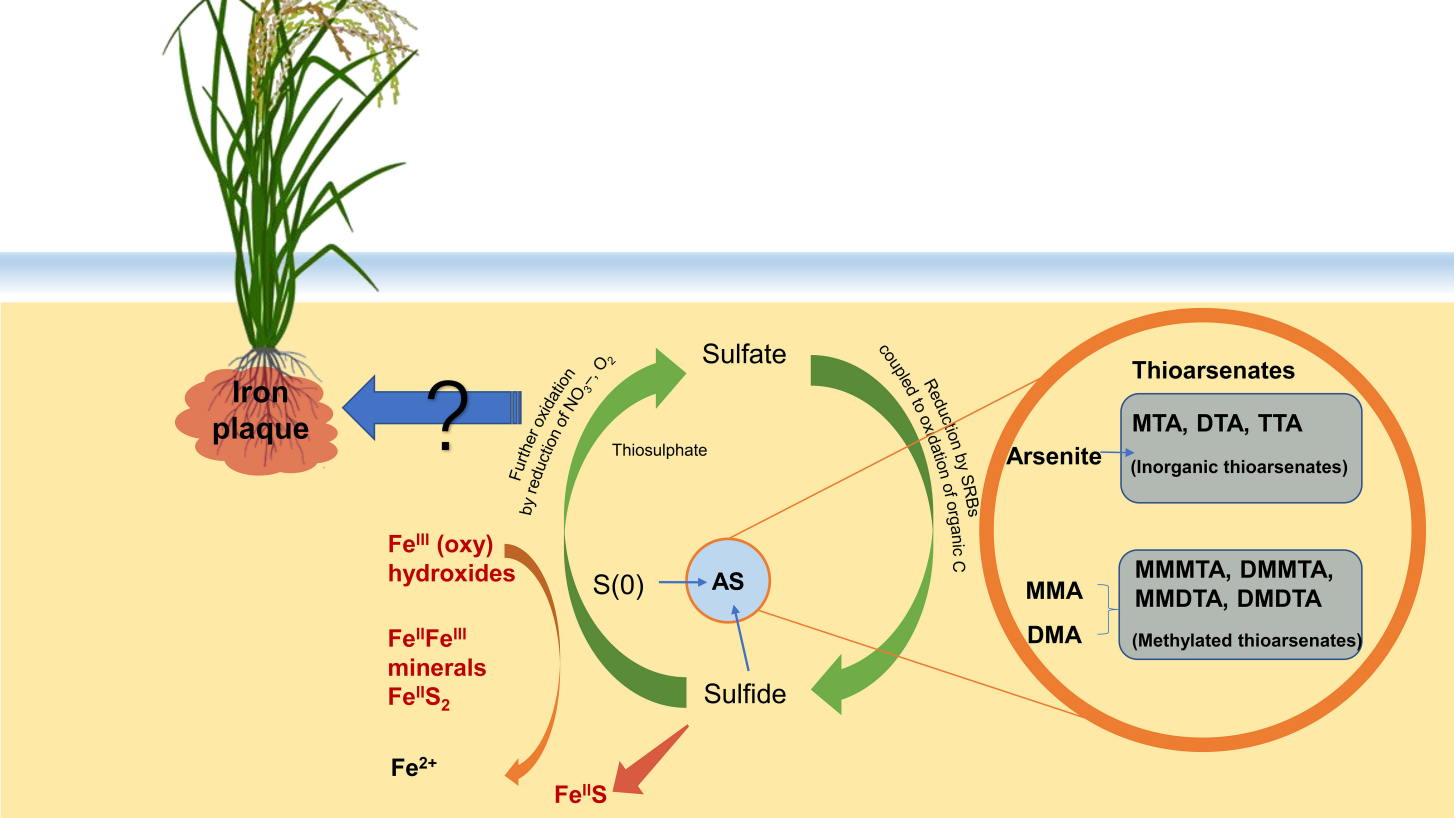
Arsenic biocycle coupling with sulfur cycle in soil. Extra SO_4_^2-^ helps mitigate As dissolution in reducing (flooding) and oxidizing (drying) and As concentration (mainly DMA and AsIII) in rice grain. Decreasing porewater AsIII (and total As) and grain methylated As in rice are along with the decline of porewater Fe, which is due to sulfate reduction and the precipitation of AsIII, as arsenic sulfide (As_2_S_3_), and iron sulfide (Fe_2_S_3_) minerals. The reduction process facilitates the coprecipitation of secondary Fe sulfide or As sulfide minerals with As. Further, sulfide could be re-oxidated to zero-valent S, which is coupled to the reduction of ferric (oxy)hydroxides and formation of mixed FeII/FeIII minerals or pyrite (FeS_2_) besides Fe^2+^. Sulfide oxidation to thiosulphate and sulfate is coupled to nitrate or oxygen reduction. The effect of S on Fe plaque formation and the fixation of As is still controversial.

Applying sulfate (SO_4_^2−^) fertilizer could decrease As concentration in both soil and rice plants, so alleviate straighthead symptoms (**Figure 3**). Regardless of reducing (flooding) and oxidizing (drying) conditions, an extra SO_4_^2−^ in the paddies helps reduce the As concentration (mainly DMA and AsIII) in rice (Hashimoto and Kanke, 2018). For example, sulfate addition significantly decreases the total As and AsIII contents by 62% and 79% under continuous flooding and by 50% and 76% under intermittent flooding, respectively, in rice grains (Liu et al., 2021). Sulfate reduction and the precipitation of AsIII as arsenic sulfide (As_2_S_3_), and iron sulfide (Fe_2_S_3_) minerals lead to a decrease of porewater AsIII (and total As) and grain methylated As in rice along with the decline of porewater Fe (Fang et al., 2023; Yan et al., 2022). Under anaerobic conditions, the reductive process facilitates the coprecipitation of secondary Fe sulfide or As sulfide minerals with As, which obstructs the reduction and dissolution of Fe (Kumarathilaka et al., 2018). Sulfide could be re-oxidized to zero-valent S, coupled to reduced ferric (oxy)hydroxides, and formed of mixed FeII/FeIII minerals or pyrite (FeS_2_) besides Fe^2+^. Sulfide oxidation to thiosulfate couples with nitrate or oxygen reduction. Such an S cycle sustains high sulfate reduction rates in the bulk soil, especially in the rhizosphere (**Figure 3**). The process is tightly associated with pH and the precursors in anaerobic soil, where inorganic thioarsenates occur predominantly at soil (pH > 6.5) and in the presence of zero-valent sulfur; methylated thioarsenates occur predominantly at soil (pH < 7) and in the presence of their precursors (methylated oxyarsenates) (Wang et al., 2020b). High concentrations of dissolved iron restrict As thiolation. Sulfate fertilization increases thioarsenate formation. Moreover, sulfate-reducing bacteria (SRB) play an important role in the reduction of sulfate to sulfide through oxidizing organics, and subsequently, the sulfurization of organic matter forms organic thiol groups (As-SH) to accomplish the immobilization of As (Burton et al., 2014). Therefore, sulfate addition indirectly prevents As dissolution from As-containing Fe minerals.

The effect of SO_4_^2−^ on Fe plaque formation and its fixation of As is still controversial, although it is well known that Fe plaque on the surface of rice roots works as a filter of As uptake, and a mixture of ferric and aluminum sulfate or feralum has been adopted as a straighthead control practice (Sara et al., 2023). Some studies suggest that sulfate addition promotes the formation of Fe plaque (Cao et al., 2018). The addition of sulfate significantly reduces the As concentration in the soil solution. However, others indicate that sulfate impairs Fe plaque and inhibits its binding to As (Fan et al., 2010; Liu et al., 2021). Excessive sulfate prevents the transfer of As from the roots to aboveground parts and reduces the proportion of AsIII in rice grains, although the effect of S on Fe plaque and the fixation of As are not always consistent.

### 3.3 Nitrogen fertilizer

High nitrogen fertilizer helps reduce the severity of straighthead (Batten et al., 2006; Dunn et al., 2006). High-N application improves plant growth and vigor and consequently increases the uptake and accumulation of S, Pi, K, Mg, Cu, Mn, and Zn at panicle initiation, thus decreasing the straighthead sterility and achieving a high yield of grains (Dunn et al., 2006).

**Figure 4:**
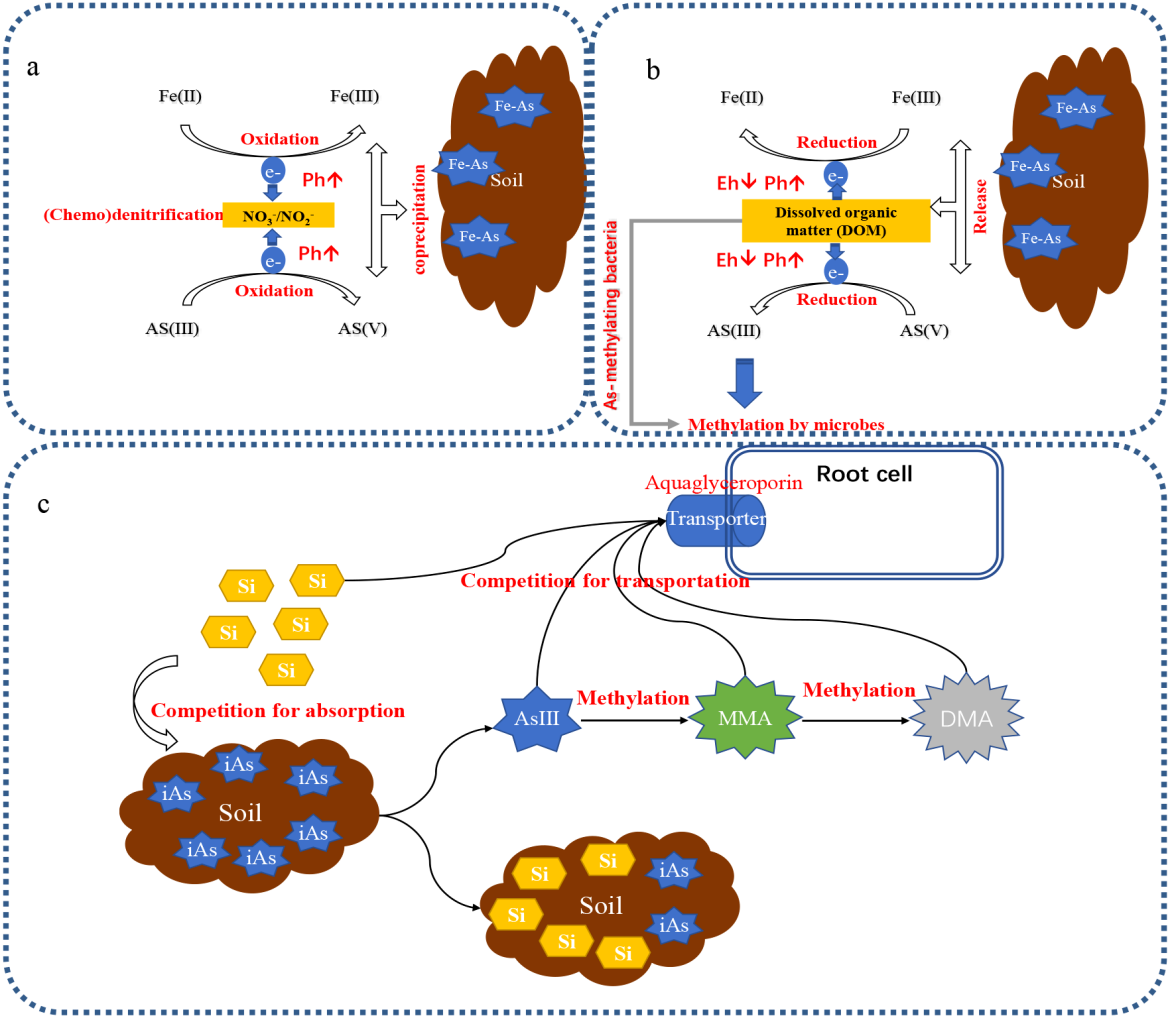
Effects of nitrogen fertilizer, dissolved organic matter (DOM), and silicon (Si) fertilizer on the availability of arsenic (As). **(a)** NO_2_^−^ reduces FeII concentration in soil solution by inhibiting FeIII reduction and enhancing nitrate-dependent FeII oxidation. The decrease of FeII concentration leads to As coprecipitation with FeIII minerals in the soil. Chemodenitrification is the subsequent abiotic reactions of FeII oxidation and NO_2_^–^ reduction at the interface of anoxic condition, which leads to the formation of secondary iron (Fe)-bearing minerals. During chemodenitrification, AsV forms monodentate and bidentate complexes with Fe minerals, and is incorporated into the structure of Fe minerals, resulting in the attenuation of As within soil and water; **(b)** DOM provides an effective source of carbon and nitrogen for microorganisms, and subsequently enhances a number of As-methylating bacteria in soil microbial community of paddy soils. The negatively charged DOM in soil acts as an electron donor or acceptor in redox reactions, which allows the reduction of AsV and FeIII, resulting in the release of As and Fe in soil solution. DOM-mediated change in Eh and pH condition can also indirectly affect the adsorption affinities of solid-phase As in paddy soil. Soil Eh potentials drop whereas pH has a tendency to increase owing to the consumption of H^+^, which promotes the release of soil As into solution; **(c)** Si restricts grain AsIII/DMA through competition for root uptake and downregulation of root Si transporters. Additionally, Si fertilizer increases AsIII/DMA concentrations in porewaters. The silicic acid competes with AsIII/DMA for binding sites on mineral phases in paddy soils to desorb iAs into porewater, or/and Si-induced desorption of iAs is subject to methylation by microbes.

Nitrate (NO_3_^−^) affects the fate of As in soil systems. Nitrate (NO_3_^−^)–dependent stimulation of FeII oxidation and/or inhibition of FeIII reduction in the bulk soil sequesters mobile As in the soil, resulting in reduced As uptake by rice (Chen et al., 2008). Under flooding conditions, As is released because of FeIII reduction when O_2_ is consumed in the paddy by aerobic bacteria and/or chemical oxidation processes, while under drying conditions, AsV is immobilized by the newly formed Fe minerals through NO_3_^−^ denitrification (**Figure 4a**). During the process, nitrate is the first alternative electron acceptor, and FeIII reduction is inhibited. Nitrite (NO_2_^−^) is an intermediary compound formed during denitrification. NO_2_^–^ also reduces FeII concentration in soil solution by inhibiting FeIII reduction, which leads to As coprecipitation with FeIII minerals in the soil (Hu et al., 2022; Zhang et al., 2017) (**Figure 4a**). Chemodenitrification is the subsequent abiotic reactions of FeII oxidation and NO_2_^–^ reduction at the interface of anoxic conditions, which leads to the formation of secondary iron (Fe)-bearing minerals (Melton et al., 2014) (**Figure 4a**). During chemodenitrification, AsV can form monodentate and bidentate complexes with Fe minerals and may even be incorporated into the structure of Fe minerals, resulting in the attenuation of As within soil and water (Smith et al., 2017). The increase of pH by chemodenitrification promotes the immobilization of AsV in newly formed crystalline Fe minerals (Hu et al., 2022). Therefore, adding NO_3_^–^ to a paddy inhibits the release of As into the soil solution.

Unlike NO_3_^−^ and NO_2_^−^, NH_4_Cl inhibits the release of As into porewater through the decrease in the pH of porewater by the release of protons through hydrolysis (Liu et al., 2022). Reducing soil pH can increase the positive charge on the surface of Fe minerals and promote the adsorption of AsV, leading to As immobilization, thus decreasing the As concentration in porewater (Di Iorio et al., 2018; Komarek et al., 2013). Unlike the As immobilization induced by NO_2_^−^/NO_3_^−^, NH_4_Cl immobilizes As over the long term, and a high-dosage application is more suitable for inhibiting the As release (Liu et al., 2022). NH_4_Cl addition does not significantly increase the Fe concentration in porewater but remarkably inhibits the release of As into porewater (Liu et al., 2022). On the contrary, some studies reported that application of ammonia N promotes the release of Fe and As into soil porewater, which is attributed to anammox (anaerobic ammonium oxidation) coupled with the FeIII reduction and release of absorbed As under anaerobic conditions (Huang and Jaffe, 2018; Weng et al., 2017; Xiu et al., 2020; Zhang et al., 2021c). Also, the addition of ammonia N decreases soil pH, which further drives the anammox process coupled with Fe(III) reduction and enhances As reduction in flooded paddy soils where anoxic conditions prevail. It has been a common consensus that both NH_4_^+^ and NO_3_^−^ fertilizers effectively reduce the As accumulation in rice grains, although whether NH_4_^+^ inhibits the release of As into soil porewater is still controversial.

### 3.4 Organic matter

Organic matter incorporation accelerates the methylation and mobilization of As in the paddy, resulting in an increased accumulation of methylated As in rice plants, contributing to the occurrence of straighthead disease (**Figure 4b**). The degree of As volatilization from soil amended with composted cereal straw is twice that obtained upon alfalfa compost supplementation (Huang et al., 2012). Dissolved organic matter (DOM) provides an effective source of carbon and nitrogen for microorganisms and subsequently enhances the number of As-methylating bacteria in the microbial community in the paddy (Goldberg et al., 2017; Li et al., 2019; Yan et al., 2020). Biogas slurry (BGS) tremendously increases As accumulation in rice plants, especially for methylated As species (Jia et al., 2013b). The concentrations of methylated As species in rice husks and grains are increased by 105.8%–105.9% and 99.7%–112.2%, respectively. Moreover, DOM considerably increases methylated As proportions more than iAs in manure-treated soils (Afroz et al., 2019). Also, DOM derived from composted pig manure or rice straw leads to an increase of four to 10 folds in As methylation and the number of As methylation-inducing bacteria, such as *Proteobacteria, Bacteroidetes, Geobacter, Sphingomonas, Streptomyces*, and *Rhodopseudomonas* in paddy soils (Yan et al., 2020).

Adding organic matter regulates microbial activities in soil by providing an energy source and depleting oxygen, which drives soils to be more reduced in favor of AsIII release and its methylation (Jia et al., 2013b). The abundance and diversity of microorganisms upon adding rice straw are reduced, whereas the prevalence of Rikenellaceae increases, resulting in high levels of methylated As present in both soil porewater and rice husks (Liu et al., 2023). Rikenellaceae is the major contributor to As methylation in straw-amended soil by reducing humics (particularly fulvic acid) and providing an electron for As reduction and release (Qiao et al., 2019). Also, rice straw amendment inhibits methanogenesis and results in MMA and DMA accumulation by suppressing DMA demethylation (Zhai et al., 2023). The demethylation of DMA is driven by methylotrophic methanogens, that is, *Methanomassiliicoccus* and *Methanosarcina*, which use the methyl group in DMA as a substrate to produce CH_4_ consequently (Chen et al., 2019).

The functional groups on the surface of DOM, such as carboxyls and hydroxyls, can directly perform ligand exchange with iAs, enhancing As mobility (Mohapatra et al., 2007; Zhang et al., 2018). The negatively charged DOM in soil acts as an electron donor in redox reactions, which reduces AsV and FeIII, releasing As and Fe in soil solution (Dong et al., 2014; Islam et al., 2004). DOM-mediated change in Eh and pH can also indirectly affect the adsorption affinities of solid-phase As in paddy soil (Redman et al., 2002). Soil Eh potentials drop, whereas pH tends to increase owing to the consumption of H^+^, which promotes the release of soil As into solution (Bennett et al., 2012). For example, adding pig manure DOM to soil significantly decreases the Eh but increases the pH, which promotes the release of solid-phase As from the soil to the solution and provides more substrates for As methylation in paddy soil (Yan et al., 2020).

Rice straw appears to have a high ability to accumulate As by absorption from paddy soils and ample production of As methylation (Zhang et al., 2021a; Zhao and Wang, 2020). Firstly, incorporating rice straw derived from As-contaminated soils further elevates the total and methylated As concentration in the soil solution and rice plants, which worsens straighthead disease. Secondly, the decomposition of straw can lead to the production of organic acids, which may lower soil pH and increase the solubility of As. This can result in higher As availability in the soil solution, facilitating its uptake by plants (Chen et al., 2022). Straw retention can create more reducing conditions in the soil, which may transform As from less mobile forms (e.g., AsV) to more mobile forms (e.g., AsIII), thereby increasing its mobility and potential for uptake (Zhang et al., 2021b). Thus, straw retention is a potential input pathway for As in agroecosystems (Yi et al., 2018). However, when DOM is at a low rate, total As in soil solution decreases to some extent (Avneri-Katz et al., 2017). This may be due to the direct adsorption of DOM by soil colloid, which compromises the contribution of ligand exchange with iAs to As release from the soil. The application of organic matter, particularly rice straw, exacerbates straighthead disease in rice plants by increasing the methylation and mobilization of arsenic. Therefore, it does not help lessen the disease but contributes to its severity.

### 3.5 Silicate fertilizer

Applying silicate (Si) fertilizer alleviates straighthead disease, thus increasing grain yield by 14.9%–58.1% (Gao et al., 2023). Similarly, in a hydroponic study, Si alleviates straighthead disease induced by DMA and MMA (Limmer et al., 2018). Also, increasing Si significantly decreases AsIII/DMA concentrations in plant roots, straws, husks, and grains (Gao et al., 2023; Limmer et al., 2018). Si restricts the uptake of AsIII/DMA by competing for root absorption and reducing root Si transporters, particularly at late stages of growth when Si uptake increases up to the most (Gao et al., 2023) (**Figure 4c**). This is because both Si and AsIII/DMA are acquired by the same transporter, OsNIP2, Lsi1 in rice plants (Li et al., 2009b), where Lsi1 expression is downregulated by Si (Gao et al., 2023; Mitani-Ueno et al., 2016). Moreover, Si decreases total As concentration in the root cell walls and xylem sap, restrains As translocation from root to shoot (Gao et al., 2021), and protects the cell wall metabolism from DMA toxicity (Tang et al., 2020).

Si fertilizer increases AsIII/DMA concentrations in porewaters (Gao et al., 2023) because the silicic acid competes with AsIII/DMA for binding sites on mineral phases in paddy soils to desorb iAs into porewater, and/or Si-induced desorption of iAs is subject to methylation by microbes (Dykes et al., 2020; Kersten and Daus, 2015). Thus, applying Si fertilizer produces two opposite effects: increasing DMA concentration in soil porewater and suppressing DMA uptake by rice plants. Some studies have demonstrated that DMA accumulation increases in rice grains, but no straighthead occurs (Li et al., 2009b; Liu et al., 2014). This suggests that the positive effect of Si on DMA availability in soil outweighs the negative effect of Si on DMA uptake (**Figure 4c**). Other studies show that the impact of Si on DMA concentrations in both husks and grains of the diseased panicles is greater than those of the typical panicles, indicating that the Si impact is enhanced by the occurrence of straighthead disease (Gao et al., 2023; Limmer et al., 2018).

### 3.6 Other minerals

The appropriate addition of phosphorus (Pi) can effectively inhibit As uptake by plants. AsV is an analog of Pi and hitches Pi-transporters in plants; thus, AsV uptake is regulated by the Pi signaling pathway (Chen et al., 2005; Kamiya et al., 2013). However, excessive application of Pi fertilizer may promote the release of Fe (hydro)oxides-associated As from soil solids into pore water due to competition between Pi and As for binding sites (Bolan et al., 2013; Lee et al., 2016). The total As concentration in pore water under low Pi dose treatments was approximately 12.5%–14.2% lower than that in normal Pi treatments during the rice tilling and heading stages (Yang et al., 2020). Similarly, phosphate-solubilizing bacteria (PSB), such as *Bacillus licheniformis* and *Pantoea dispersa*, can reduce dependency on chemical Pi fertilizers by 50%, while simultaneously lowering As bioavailability through competitive inhibition and the formation of Fe plaques on roots (Irshad et al., 2022; Rawat et al., 2022). Conversely, Pi deficiency exacerbates As toxicity by upregulating transporters, which inadvertently increases the assimilation of AsIII and AsV (Wang et al., 2022). Collectively, the application of Pi exhibits a dual effect on As in the soil–plant system: appropriate addition of Pi can inhibit plant uptake of As by regulating the Pi signaling pathway and via the action of phosphate-solubilizing bacteria (PSB), while excessive Pi may promote the release of soil-bound As into pore water, and Pi deficiency can exacerbate As toxicity by upregulating transporters. Thus, rational management of Pi input is crucial for balancing As reduction and soil–plant health.

Zinc oxide nanoparticles (ZnONPs) and zinc ions (Zn^2+^) both significantly reduced the total As in rice roots and shoots, primarily by decreasing the levels of inorganic As(III) and organic As species (Ma et al., 2020). This reduction can be attributed to several key mechanisms. Firstly, Zn competes with As for uptake sites on the root cell membrane, leading to competitive inhibition and reduced As absorption (Gong et al., 2020). Secondly, Zn reacts with As in the soil to form insoluble Zn-As complexes, which are less bioavailable and less likely to be absorbed by plant roots (Das et al., 2016). Additionally, Zn enhances the activity of antioxidant enzymes such as superoxide dismutase (SOD), peroxidase (POD), and catalase (CAT), which help scavenge reactive oxygen species (ROS) generated during As toxicity (Jalil et al., 2024; Yan et al., 2021). Furthermore, Zn upregulates the expression of enzymes involved in As defense and detoxification pathways, for example, ascorbic acid (AsA)—glutathione (GSH) cycle enzymes (Zeeshan et al., 2021). This regulation prevents the accumulation of H_2_O_2_ in tissues (Foyer and Noctor, 2011), thereby alleviating arsenic toxicity. Lastly, Zn improves overall plant health and nutrient homeostasis, enhancing the plant’s ability to resist As toxicity (Wu et al., 2020). These combined mechanisms effectively mitigate As uptake and toxicity in rice plants, attenuating straighthead symptoms.

Adding Cu has been effective in reducing straighthead. In highly anaerobic soils, H_2_S and high organic matter may significantly reduce Cu solubility. Decreasing Cu bioavailability results in its deficiency and leads to straighthead development (Belefant-Miller and Beaty, 2007).

Ca supplementation improves the tolerance of rice seedlings to As-induced oxidative stress by reducing As uptake, enhancing their antioxidant defense and glyoxalase systems, and improving soil condition (Rahman et al., 2015). One of the effects of Ca supplementation is the immobilization of both AsIII and AsV. The precipitates of Ca–As–O are identified when AsIII is the main form at Ca/As molar ratios ≥2.5:1, while NaCaAsO4 •7.5H_2_O is formed when AsV is the primary source of the contamination. The effectiveness of both AsIII and AsV immobilization in these slurries increases along with an increase in Ca/As molar ratios (Moon et al., 2004).

Selenate may induce a reduction of As(V) to As(III) and As(III) efflux to the external medium and significantly downregulate the expression of *OsNramp1* and *OsLsi2* associated As transport in rice, thereby decreasing As accumulation and As(III) proportion in rice (Huang et al., 2024).

Biochar-supported Fe-(oxyhydr)oxide and layered double hydroxide (FLBC) modified As species significantly reduced As in porewater and rice tissue, which is due to an increase in the Fe plaques on root surfaces (Lyu et al., 2024). The increase in Fe plaques is mainly derived from the Fe(II) sink influenced by Fe(III)-reducing bacteria and the conversion of crystalline-like FeOx to an amorphous-like structure on Fe plaque.

## 4 Genetic strategy to control straighthead

### 4.1 Resistant cultivars

Genetic advances have been helpful in managing straighthead. Many straighthead-resistant cultivars with responsible quantitative trait loci (QTL)/genes have been identified, which provides another option to control straighthead more effectively and economically than conventional cultivation practices. In our previous study, 200 Chinese cultivars were introduced in 1996, from which 19, including 18 *indica* and one *japonica* cultivar, were identified as utterly resistant to straighthead without yield loss (Yan et al., 2005). Further, 26 resistant accessions (most of them from China) were identified from a large-scale screening of 990 accessions in the U.S. Department of Agriculture (USDA) rice core collection (Yan et al., 2004). The most resistant accessions in those evaluations have been immediately used to develop genetic populations for gene search. Of cultivars from the International Rice Research Institute (IRRI) and the French Agricultural Research Centre for International Development (CIRAD), 20 are identified to be resistant to straighthead in nutrient solution containing disodium hydrogen arsenate (MSDS, Na_2_HAsO_4_) instead of MSMA (Dasgupta et al., 2004). Among these resistant cultivars above programs, most belong to *indica*, and only a few belong to *japonica*. Those resistant cultivars take up much less As than those susceptible ones by limiting As uptake and neutralizing As toxicity. Modulation of As uptake, transport, and sequestration into the vacuole dramatically reduces As accumulation in rice grain (Jia et al., 2013a).

### 4.2 Genes involved in arsenic uptake through Pi/Si transporter

AsV, a chemical analog of Pi, enters rice plants through Pi-transporters, while AsIII, an analog of boric acid and silicic acid, utilizes the Si and Lsi1 transporters (Suriyagoda et al., 2018) (**Table 1**). The rice aquaglyceroporin *Lsi1* bears much of the uptake capacity for methylated As, for example, MMA or DMA from herbicides, pesticides, or microbial As methylation, whereas *Lsi2* has little capacity to transport them (Li et al., 2009a). The Pi transporter proteins and As transcription factors work cumulatively to uptake and translocate AsV in rice plants. *OsPHF1* mutation empowers the plant with As tolerance by reducing Pi uptake and AsV accumulation by interfering with the membrane location of two Pi-transporters, OsPT2, and OsPT8 (Chen et al., 2011). In contrast, the overexpression of *OsPHR2* can enhance Pi and AsV uptakes from roots and their translocation from roots to shoots (Chen et al., 2011). The induction of *OsPT2*, *OsPT4*, and *OsPT8* by Pi deficiency is drastically suppressed by AsV (Kamiya et al., 2013), suggesting that the AsV can interact with the Pi regulatory machinery. Although OsPT1 is not induced by Pi deficiency, it is involved in AsV uptake from soil or apoplast, leading to As accumulation in shoots (Kamiya et al., 2013). In contrast, the mutants of *OsPT1*, *OsPT4*, and *OsPT8* all take much less As, thus accumulating less As than their wild type (Cao et al., 2017; Kamiya et al., 2013; Wang et al., 2016). OsIPS1, a key regulator of the Pi-signaling pathway, can be suppressed by AsV (Hou et al., 2005). PHO1, a Pi transporter responsible for the xylem loading of Pi, plays a significant role in loading AsV into the xylem of plants (Kamiya et al., 2013). Moreover, the transcription factors of OsWRKY28 and OsPHR2 and Pi transporter traffic facilitator OsPHF1 all engage in the uptake of AsV (Chen et al., 2011; Wang et al., 2018).

**Table 1:** Genes involved in various functions of metabolism on arsenic (As) in rice.

Metabolism	Gene name	Category	Function	As species	Reference
Phosphate transport	*OsPHT1;1* (*OsPT1*)	Phosphate transporter	Pi/As uptake and translocation root to shoot	AsV	(Kamiya et al., 2013)
pathway	*OsPht1;2* (*OsPT2*)	Phosphate transporter	Pi/As uptake and translocation root to shoot	AsV	(Chen et al., 2011; Kamiya et al., 2013)
	*OsPht1;4; OsPT4*	Phosphate transporter	Pi/As uptake and translocation root to shoot	AsV	(Kamiya et al., 2013)
	*OsPht1;8; OsPT8*	Phosphate transporter	Pi/As uptake and translocation root to shoot	AsV	(Chen et al., 2011)
	*OsPHF1*	Phosphate transporter traffic facilitator1	Pi/As Transportation	AsV	(Chen et al., 2011)
	*OsPHR2*	MYB-domain transcription factor; PHOSPHATE RESPONSE1 ortholog	Pi/As-signaling regulator	AsV	(Wu and Wang, 2011)
	*OsWRKY28*	WRKY transcription factor	Pi/As accumulation in the shoots	AsV	(Wang et al., 2018)
	*OsIPS1*	a member of TPSI1/Mt4 family	A key regulator of Pi deficiency signaling	AsV	(Hou et al., 2005)
	*OsPHO1*	Phosphate transporter	xylem loading of Pi/As to the shoots	AsV	(Kamiya et al., 2013)
Aquaglyceroporin transport	*OsNIP1;1*	Aquaglyceroporin transporter	Pi/As Transport	AsIII	(Isayenkov and Maathuis, 2008; Sun et al., 2018)
pathway	*OsNIP2;1 (Lsi1)*	Aquaglyceroporin transporter	Pi/As Transport	AsIII and methylated As	(Isayenkov and Maathuis, 2008)
	*OsNIP2;2 (Lsi6)*	Aquaglyceroporin transporter	Pi/As Transport	AsIII	(Isayenkov and Maathuis, 2008)
	*OsNIP3;1*	Aquaglyceroporin transporter	Pi/As Transport	AsIII	(Isayenkov and Maathuis, 2008)
	*OsNIP3;2*	Aquaglyceroporin transporter	Pi/As Transport	AsIII	(Katsuhara et al., 2014)
	*OsNIP3;3*	Aquaglyceroporin transporter (Si transporter)	Pi/As Transport	AsIII,	(Katsuhara et al., 2014; Sun et al., 2018)
	*OsLsi2*	Aquaglyceroporin transporter	Pi/As Transport	AsIII	(Isayenkov and Maathuis, 2008)
Vacuolar sequestration	*OsNRAMP1*	NRAMP transporter	Mediated As/Cd xylem loading	AsIII	(Tiwari et al., 2014)
	*OsACR2.1*	Arsenate reductase	Reduction of AsV to AsIII	AsV	(Duan et al., 2007)
	*OsACR2.2*	Arsenate reductase	Reduction of AsV to AsIII	AsV	(Duan et al., 2007)
	*OsHAC1;1*	Arsenate reductase	Reduction of AsV to AsIII	AsV	(Shi et al., 2016)
	*OsHAC1;2*	Arsenate reductase	Reduction of AsV to AsIII	AsV	(Shi et al., 2016)
	*OsHAC4*	Arsenate reductase	Reduction of AsV to AsIII	AsV	(Xu et al., 2017)
	*OsGrx*	Arsenate reductase	GSH-dependent arsenate reductase (AR) for reducing AsV to AsIII	AsV	(Dubey et al., 2016)
	*OsABCC1*	ABC transporter; Arsenate reductase	Transportation of AsIII-PC complexes to the vacuole; Reduction of AsV to AsIII	AsIII/AsV	(Song et al., 2014)
	*OsCLT1*	CLT1 transporter; Arsenate reductase	Transportation of AsIII-PC; Reduction of AsV to AsIII	AsIII/AsV	(Yang et al., 2016)
	*OASTL-A1*	O-acetylserine(thiol)lyase	Cysteine biosynthesis for As translocation	AsV	(Wang et al., 2020a)
	*OsPCS1*	Phytochelatin synthase	Phytochelatins synthesis for AsIII-PC complexes	AsIII	(Hayashi et al., 2017)
	*ATT1 (OsNPF5.8)*	Nitrate transporter/peptide transporter	Regulating the expression of As transporters and PC-synthases	AsIII	(Lim et al., 2020)
	*OsARM1*	A transcription factor	Regulating the expression of OsLsi1, OsLsi2, and OsLsi6. OsLsi2	AsIII	(Chen et al., 2015)

**Figure 5:**
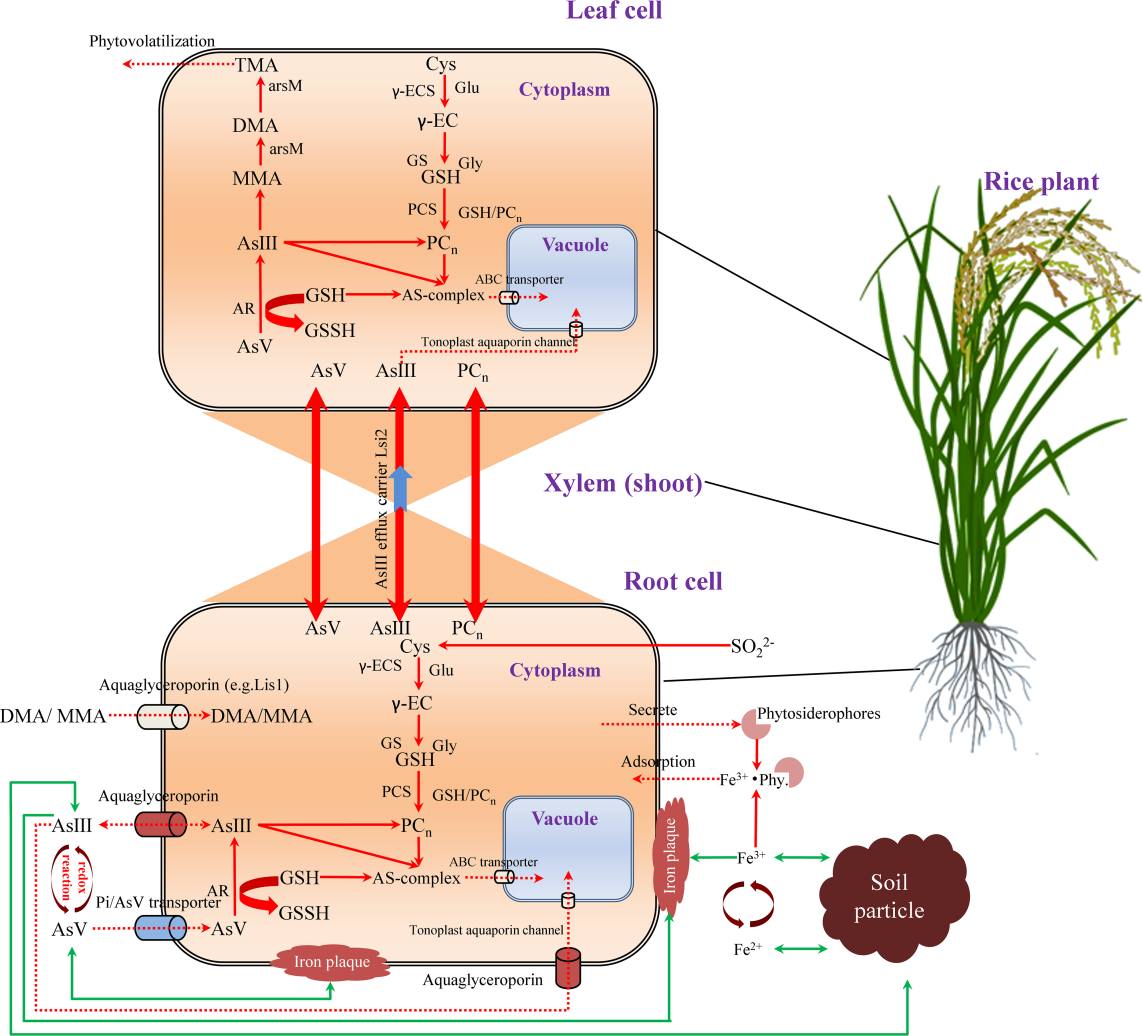
Transportation diagram on arsenic from soil to root and up to leaf in rice. AsV and AsIII influx into a root cell through phosphate transporters and aquaglyceroporins, respectively, whereas a small amount of organic As (MMA and DMA) enters through aquaglyceroporins, for example, Lsi1. Plants assimilate SO_4_^2−^ to form Cys for the synthesis of GSH in two ATP-dependent steps, where γ-EC is synthesized by γ-ECS using Cys and γ-Glu as substrates, and GSH is synthesized by GS, using γ-EC and Gly as substrates. In response to As, plants induce PCn synthesis through PCS enzyme. PCn can be transported from root to shoot and vice versa. During As-detoxification in the cytoplasm, the majority of AsV is reduced to AsIII by AR. AsIII can be then complexed with GSH or PCs through the sulfhydryl group of Cys. The AsIII-PCs complex or free AsIII can be transported across the tonoplast via an ABC transporter to be sequestered in the vacuole. AsIII is a main species and transported from the roots to various organs above the ground via an efflux protein, Lsi2. Volatile TMA is the final product from the As methylation pathway, which can be volatilized from the plant. External AsV, that has a high affinity for Fe-plaque on the root surface, reacts with FeIII to yield the highly insoluble iron arsenate. In the rhizosphere, Fe-plaque on the root surface is derived from precipitated Fe oxides. The phytosiderophores excreted from rice roots play an important role in Fe acquisition of plant. Green lines with arrow indicate adsorption to soil particles. Red dashed lines with arrows show active plant transport processes. ABC, ATP-binding cassette transporter; AsIII, arsenite; AsV, arsenate; AR, arsenate reductase; Cys, cysteine; DMA, dimethylarsinic acid; Fe^2+^, ferrite; Fe^3+^, Ferrate; Gly, glycine; GS, glutathione synthetase; GSH, glutathione; MMA, monomethylarsonic acid; PCn, phytochelatins as the polymers of GSH; PCs, phytochelatins; PCS, phytochelatin synthase; TMA, trimethylarsine; TMAO, trimethylarsine oxide; SO_4_^2−^, sulfate; γ-EC, γ-glutamylcysteine; γ-ECS, γ-glutamylcysteine synthetase; γ-Glu, γ-glutamic acid.

AsIII uptake from the soil takes place by different plasma membrane intrinsic proteins (MIP) and nodulin 26-like intrinsic protein (NIP) aquaglyceroporins (Mosa et al., 2012) (**Table 1**). In rice, two transporters of the NIP subfamily, Lsi1 (NIP2;1) and Lsi2, are also permeable to AsIII (Isayenkov and Maathuis, 2008) (**Figure 5**). Lsi1 pumps AsIII to the outside of the cell when the internal AsIII concentration is higher than that of the outside, while Lsi2 transports both Si and AsIII from exodermal and endodermal cells to the xylem stele (Isayenkov and Maathuis, 2008). Lsi2 has a much greater impact on AsIII accumulation in shoots and grains than Lsi1 (Isayenkov and Maathuis, 2008). The other four NIP proteins, OsNIP2;2 (Lsi6), OsNIP3;1, OsNIP3;2, and OsNIP3;3, are also permeable to AsIII when expressed in *Xenopus laevis* oocytes (Isayenkov and Maathuis, 2008; Katsuhara et al., 2014). OsNRAMP1 on the plasma membrane of endodermis and pericycle cells in the xylem may mediate loading and enhance the accumulation of As and Cd by assisting their loading in roots up to shoots (Tiwari et al., 2014). A transcription factor, OsARM1 (ARSENITE-RESPONSIVE MYB1), is involved in the uptake and translocation of AsIII by regulating the expression of OsLsi1, OsLsi2, and OsLsi6, which in turn influences As accumulation in nodes and grains of rice (Chen et al., 2015). Together, rice plants employ diverse transporters and regulatory mechanisms for the uptake and translocation of AsV and AsIII, with Pi transporters, MIP, and NIP, while mutations or expression changes of related genes can affect As accumulation and tolerance in rice.

**Table 2:** QTL responsible for straighthead resistance, arsenic accumulation or tolerance, and Si concentration in rice.

Trait	Chr.	QTL^b^	Phenotypic variation explained (%)	Allele effect	References	Neighbor QTL	References
Straighthead^a^	Chr.2	*qSH-2*	11.10	−0.90	(Li et al., 2016)	Pi-uptake QTL	(Wissuwa et al., 1998)
						*AsS*	(Zhang et al., 2008)
						*qAsS2*	(Murugaiyan et al., 2019)
	Chr.3	*qSH-3*	18.00	0.70	(Bennett et al., 2012)	*AsR*	(Zhang et al., 2008)
	Chr.6	*qSH-6*	13.00	−21.90		Pi-uptake QTL	(Wissuwa et al., 1998)
						*AsTol*	(Dasgupta et al., 2004)
						*AsSe1*	(Zhang et al., 2008)
						*qAsS6*	(Murugaiyan et al., 2019)
	Chr.7	*qSH-7*	12.00	−21.70	(Bennett et al., 2012)		
	Chr.8	*qSH-8^b^*	28.10	−1.14	(Li et al., 2016)	*AsSe2*	(Zhang et al., 2008)
						*PS1*(Pi-uptake QTL)	(Zhang et al., 2008)
		*qSH-8^c^*	46.00	−22.10	(Bennett et al., 2012)	*qAsR8.1*	(Murugaiyan et al., 2019)
		*qSH-8^d^*	67.00	−22.10		*qAsR8.2*	
	Chr.11	*qSH-11*	8.00	−21.10			
As-accumulation	Chr.2	*AsS*	24.40	−1.38	(Zhang et al., 2008)	Pi-uptake QTL	(Wissuwa et al., 1998)
		*qAsS2*	9.18–9.82	−1.09 to −1.13	(Murugaiyan et al., 2019)	*qSH-2*	(Li et al., 2016)
	Chr.3	*AsR*	18.20	11.18	(Zhang et al., 2008)	*qSH-3*	(Bennett et al., 2012)
	Chr.5	*qAsS5.1*	3.72–4.12	−1.05 to −1.16	(Murugaiyan et al., 2019)		
		*qAsS5.2*	4.03–5.66	−1.36 to −1.09			
	Chr.6	*AsSe1*	26.30	−0.081		Pi-uptake QTL	(Wissuwa et al., 1998)
		*qAsS6*	3.58–5.32	−1.49 to −1.28	(Murugaiyan et al., 2019)	*AsTol*	(Dasgupta et al., 2004)
						*qSH-6*	(Bennett et al., 2012)
	Chr.8	*AsSe2*	35.20	−0.092		*PS1*	(Zhang et al., 2008)
		*qAsR8.1*	10.51	−11.79	(Murugaiyan et al., 2019)	*qSH-8*	(Bennett et al., 2012; Li et al., 2016)
		*qAsR8.2*	8.72	−10.67			
	Chr.9	*qAsS9.1*	18.37 –19.20	−1.09 to −1.06	(Murugaiyan et al., 2019)		
		*qAsS9.2*	20.59–21.35	−1.13 to −1.04			
As-tolerance	Chr.6	*AsTol*			(Dasgupta et al., 2004)	Pi-uptake QTL	(Wissuwa et al., 1998)
						*AsSe1*	(Zhang et al., 2008)
						*qSH-6*	(Bennett et al., 2012)
Hull-Si concentration	Chr.1	*qSi1-1*		29.55	(Bryant et al., 2011; Pinson et al., 2022)	*StHD*	(Pinson et al., 2022)
		*qSi1-2*		−25.32	(Pinson et al., 2022; Wu et al., 2017)	*StHD* *As-QTL*	(Pinson et al., 2022)
		*qSi1-3*		26.80	(Pinson et al., 2022)	*StHD* *As-QTL*	(Pinson et al., 2022)
	Chr.2	*qSi2-1*		−22.13	(Pinson et al., 2022)	*StHD* *qSH-2*	(Pinson et al., 2022)
		*qSi2-2*		27.78	(Bryant et al., 2011; Pinson et al., 2022)*i*	*StHD*	(Li et al., 2016; Pinson et al., 2022)
	Chr.5	*qSi5-2*		21.78	(Bryant et al., 2011; Pinson et al., 2022)	*StHD*	(Pinson et al., 2022)
	Chr.6	*qSi6-3*		40.20	(Bryant et al., 2011; Pinson et al., 2022)	*StHD*	(Pinson et al., 2022)
	Chr.7	*qSi7-3*		−22.00	(Bryant et al., 2011; Pinson et al., 2022; Wu et al., 2017)	*StHD*	(Pinson et al., 2022)
	Chr.8	*aSi8-1*		−26.40	(Pinson et al., 2022)	*StHD*	(Pinson et al., 2022)
	Chr.10	*qSi10-1*		−33.30	(Pinson et al., 2022; Wu et al., 2006)	*StHD*	

a Straighthead was induced by monosodium ethane arsenate incorporated into the soil and rated by seed set and plant growth.

As-accumulation in roots, shoots and grains was measured by an atomic fluorescence spectrometry at the seedling stage and mature stage.

As-tolerance was determined by measuring root growth in the experimental condition, where the germinating seeds were floated on alkathene beads filled with either phosphate-free nutrient solution or the same nutrient solution supplemented with di-sodium hydrogen arsenate.

*qSH*/*StHD*, QTL related to straighthead; *AsS*, QTL related to As concentrations in shoots; *AsR*, QTL related to As concentrations in roots; AsSe, QTL related to As concentrations in brown rice; *AsTol*: QTL related to As tolerance *qSH-2* and *qSH-8*

b were both identified in the F_2_ and F_2:3_ population derived from across of Zhe733/R312; *qSH-3* and *qSH-8*

c in the F_9_ population of Cocodrie*/*Jing185; *qSH-6*, *qSH-7* and *qSH-8*

d in the F_9_ population of Zhe733/R312; *AsS*, *AsR*, *AsSe1*, and *AsSe2* in the double-haploid population of CJ06/TN1; *AsTol* in the F_6_ RILs of varieties Bala/Azucena. *qSi1* were all identified within the USDA Rice Mini-Core Collection.

### 4.3 Genes involved in arsenate reductase and phytochelatin

Another mechanism for plants tolerant to As is to increase the ability to detoxify AsV, including complexation with thiol compounds, GSH reduction of AsV into AsIII by arsenate reductase (AR), complexation with PC, and sequestration of the AsIII-PC complexes in the vacuole (Farooq et al., 2016) (**Figure 5**). Before loading into the xylem for translocation, AsV gets reduced to AsIII in the rice roots by AR, that is, OsHAC1;1, OsHAC1;2 (Shi et al., 2016), OsHAC4 (Xu et al., 2017), OsGrx (Dubey et al., 2016), OsACR2.1, and OsACR2.2 (Duan et al., 2007). The mutation of *OsHAC1;1* or *OsHAC1;2* decreases the reduction of AsV to AsIII in roots, reducing AsIII efflux to the external medium but increasing As accumulation in shoots, while overexpression of either *OsHAC1;1* or *OsHAC1;2* increases AsIII efflux and reduces As accumulation, which enhances AsV tolerance (Shi et al., 2016). Similarly, the mutants of As reductase (OsHAC4) exhibit a low As reduction but a high As accumulation, which is due to less efflux of AsIII outside the cell (Xu et al., 2017).

After AsIII is reduced from AsV or taken up by the root, a portion is immediately released to the rhizosphere mediated by Lsi1, a bidirectional channel (Zhao et al., 2010) (**Figure 5**). The remaining As is sequestered into root vacuoles or delivered to various organs (Zhao et al., 2010) (**Figure 5**). The GSH and PCs are greatly increased when plants are exposed to As, which empowers plants with both constitutive and adaptive tolerance to As. When exposed to As, rice immediately increases the production of PCs to reduce As transport to the grain (Batista et al., 2014). In rice, ABC transporters (OsABCC) compartmentalize the cellular AsIII by transporting it into the vacuoles. OsABCC1 is not only responsible for transporting AsIII-PC complexes to the vacuole but also involved in detoxifying and reducing As in grains. At the tonoplast in the phloem region of the nodes, OsABCC1 limits As translocation to the grains by sequestering it into the vacuoles of the companion cells in the nodes, whereas knockout of *OsABCC1* increases the sensitivity of plants to As in rice (Song et al., 2014). OsABCC1 preferentially cooperates with OsPCS1 to sequester rice, as rice has another phytochelatin synthase, OsPCS2 (Hayashi et al., 2017). The *OsPCS1* is more sensitive to activation by As than by cadmium (Cd), whereas *OsPCS2* is more insensitive to activation by As than by Cd (Yamazaki et al., 2018). *OsCLT1* affects As and Cd detoxification by mediating the export of γ-glutamylcysteine and GSH from plastids to the cytoplasm (Yang et al., 2016). *OsCLT1* mutant accumulates 50% less As in its roots than the WT but significantly increases As accumulation in its shoots due to a decrease of GSH and PC contents in the cytoplasm. OASTL-A1 is an O-acetyl serine (thiol) lyase in cysteine biosynthesis and As detoxification in rice (Wang et al., 2020a). Knockout of *OsOASTL-A1* leads to lower cysteine, GSH, and PC levels in roots but increases the sensitivity to AsV stress significantly. ATT1 (OsNPF5.8) is potentially one of the nitrate transporters/peptide transporters to be involved in As tolerance (Lim et al., 2020). Rice with ATT1 has a dramatic increase in transcription abundances of As transporters and PC-synthases for sequestrating vacuolar As. Collectively, plant tolerance to AsV involves AsV reduction by arsenate reductases, AsIII complexation with GSH/PCs, vacuolar sequestration, rhizosphere efflux, and regulation of thiol biosynthesis and transporters to limit As translocation.

### 4.4 QTL involved in As/Pi/Si uptake for controlling straighthead

In the United States, much effort has been put into unraveling the genetic mechanism of straighthead and developing its resistant cultivars for many decades. The heritability of straighthead resistance could be up to 61%, and interaction with the environment is relatively small (Rasamivelona et al., 1995). A genetic analysis of the USDA rice germplasm core collection, including Chinese cultivars, results in six DNA markers significantly associated with straighthead resistance, which explained 35% of the total phenotypic variation (Agrama and Yan, 2009). Using F_2_ and F_2:3_ populations from a cross of Zhe733 (resistant)/R312 (susceptible), two major QTL (*qSH-2* and *qSH-8*) explained 11% and 28% of the straighthead variations, respectively (Li et al., 2016) (**Table 2**). Using two F_9_ recombinant inbred line (RIL) populations, Zhe733 (resistant)/R312 (susceptible) and Cocodrie (susceptible)/Jing185 (resistant), a major QTL, *qSH-8*, within the same region, explained 46% of the total variation of straighthead in one and 67% in another population (Bennett et al., 2012). Another four QTL, *qSH-3*, *qSH-6*, *qSH-7*, and *qSH-11*, are also identified in this study, explaining 18%, 13%, 12%, and 8% of the phenotypic variation, respectively. Two markers, insertion and deletion (InDel) 11 and simple sequence repeat (SSR) AP3858-1, are so adjacent to *qSH-8* that they co-segregated with straighthead resistance in both RIL populations, as well as in a global collection of diverse accessions. A fitness test (*X* ^2^) demonstrates a high association of InDel 11 with straighthead (*P* = 0.0014), where 76.2% of the genotypes match the phenotypes among those global accessions. Similarly, AP3858–1 is highly associated with straighthead (*P* = 0.0004) with a match of 73.5%.

Some QTL for straighthead resistance are co-located with As/Si-related genes (**Table 2**). A major As-tolerant QTL (*AsTol*) (Chr. 6) for the percentage of root length in MSDS-treated/control was identified using an F_6_ RIL population of “Bala/Azucena” cross (Dasgupta et al., 2004). Another study identified four As-related QTL in a double-haploid (DH) population of CJ06 (*Japonica*)/TN1 (*Indica*) treated by AsV (Zhang et al., 2008). *AsS* (Chr. 2) for As-accumulation in shoots, *AsR* (Chr. 3) in roots, *AsSe1* (Chr. 6), and *AsSe2* (Chr. 8), both in brown rice, explain 24%, 18.2%, 26.3%, and 35.2% of the total variance, respectively. Recently, two QTL (*qAsR8.1* and *qAsR8.2*, explaining about 10% of variance) for As content in roots were mapped on chromosome 8, while six QTL (*qAsS2*, *qAsS5.1*, *qAsS5.2*, *qAsS6*, *qAsS9.1*, and *qAsS9.2*, individually explaining from 8.6% to 12.6% of the phenotypic variance) for As content in shoots were mapped on chromosomes 2, 5, 6, and 9, respectively, using a backcross population (BC_1_F_6_) of WTR1 (*indica*) and Hao-an-nong (*japonica*) treated by sodium-(meta)-arsenite (AsIII) (Murugaiyan et al., 2019). Coincidentally, the straighthead-related QTL, *qSH-6* (Chr. 6) (Bennett et al., 2012), is proximate to As-related QTL, *AsTol* (Dasgupta et al., 2004), *qAsS6* (Murugaiyan et al., 2019), and *AsSe1* (Zhang et al., 2008), where a Pi-uptake QTL is also present (Wissuwa et al., 1998)*. qSH-2* (Chr. 2) (Li et al., 2016) is adjacent to *AsS* (Zhang et al., 2008) and *qAsS2* (Murugaiyan et al., 2019), *qSH-3* (Chr. 3) to *AsR* (Zhang et al., 2008), and *qSH-8* (Chr. 8) (Bennett et al., 2012; Li et al., 2016) to *AsSe2* (Zhang et al., 2008), *qAsR8.1*, and *qAsR8.2* (Murugaiyan et al., 2019). *qSH-2* is co-located with Pi-uptake QTL (Wissuwa et al., 1998), while *qSH-8* is with *PS1* for Pi-accumulation in shoots (Zhang et al., 2008). Their co-location provides circumstantial evidence that As and Pi may share the same transporters. Moreover, QTL for grain-As and straighthead (StHD) resistance are studied within the USDA Rice Mini-Core Collection treated by MSMA, which yields the number of QTL from 9 to 33 for each of the As-associated traits (Pinson et al., 2022). For example, 10 QTL are co-associated with StHD and Si in hulls, and four QTLs are co-associated with StHD and As in grains. These StHD-related QTL are overlapped with As/Si-related QTL. These co-associations or co-localizations suggest that As uptake depends on Pi/Si transporters from roots to shoots, which are responsible for straighthead. Therefore, these QTL markers can be used for developing straighthead-resistant cultivars through marker-assisted selection (MAS).

## 5 Conclusions and future perspectives

As toxicity is believed to be one of the most important factors for straighthead disease. In particular, methylated As is highly toxic to the reproductive tissues. Thus, DMA has long been used for its evaluation. As enters rice through several pathways, a portion of the acquired As accumulates in the grain. When As accumulates in grains or other edible parts of plants, it can enter the human body through the food supply chain, potentially leading to health problems. Until now, the strategies of cultivation have been proposed to deal with straighthead, including water and fertilizer management. The optimization of water and fertilizer management increases the redox potential for As oxidation and/or changes the microbial community for As demethylation in rice fields, which results in increasing its immobility or affinity with other minerals. Additionally, some fertilizers (Pi and Si) compete with As for transporters to inhibit As entry into the rice plants.

In water management, drying a field during plant growth for straighthead control can also stress the plants, potentially decreasing grain yield. Additionally, the operation of draining, drying, and re-flooding uses extra labor and power resources, thus increasing costs for the producers. These practices are estimated to cost at least $27 million and waste 74 million cubic meters of water in Arkansas annually (Yan et al., 2014). D&D practice is applied to more than one-third of the rice acreage yearly in Arkansas as a preventative measure (Wilson, per. comm., Extension Agronomist on Rice). Thus, preserving natural water resources is important for the long-term economic viability of these countries, which are bothered by straighthead. Therefore, water management is costly for rice growers, wastes natural resources, and results in drought-related yield loss.

Fertilizer management is a very complex process. The effects of mineral fertilizer on As fixation are still controversial. For example, it is still debated whether sulfate affects Fe plaque formation and the fixation of As, whether NH_4_Cl addition increases the release of Fe and As into porewater, and what DOM concentration should be applied to reduce total As in soil solution. In other words, the effectiveness of these fertilizers on straighthead prevention greatly depends on the soil conditions. So, a test on the specific soil condition is required before applying these fertilizers. Moreover, the use of fertilizers to compete with As uptake may have unintended consequences on soil fertility and the broader environment, which require a thorough assessment to ensure sustainable agricultural practices.

The use of resistant cultivars presents a viable approach for the effective and economical control of straighthead disease. As tolerance primarily relies on the limitation of As uptake by repressing its transporters and minimizing As toxicity through mechanisms such as the reduction of AsV, complexation of AsIII, and sequestration of As in the vacuoles of roots or shoots. Genetic mapping has identified a series of QTL associated with straighthead, which are related to the accumulation and transport of Pi and silicon Si. These findings indicate that As uptake via the Pi/Si transporter is a significant contributor to the occurrence of straighthead. Therefore, breeding varieties with As tolerance represents a practical strategy to mitigate the impact of straighthead. The adoption of resistant cultivars effectively tackles the challenges of water management, such as high costs, water resource wastage, and potential yield losses. Additionally, it addresses concerns regarding fertilizer application, which is heavily reliant on soil conditions and poses significant environmental pollution risks.

It is noteworthy that other resistant varieties may possess additional mechanisms to cope with As stress. For instance, our previous research identified a significant positive correlation between the growth period of the lines and their resistance (Li et al., 2016). Specifically, lines with early heading exhibit greater resistance. The early heading may also represent a promising direction for breeding resistant varieties, as these varieties may possess alternative mechanisms to combat As stress. Over the past few decades, we have identified numerous resistant varieties that not only provide experimental materials for exploring underlying mechanisms but also offer a broad pool of resistant genes. By utilizing these resistant genes, we can introduce them into lines with other desirable agronomic traits through hybrid breeding. However, the specific mechanisms by which these resistant cultivars achieve straighthead resistance require further investigation. Additionally, a comprehensive understanding of all the processes involved in straighthead is yet to be achieved, since many other environmental factors may also cause the disease. This implies that more in-depth research is needed to fully elucidate the complex interactions between plant genetics and other environmental factors.
